# Giant invasive *Heracleum persicum*: Friend or foe of plant diversity?

**DOI:** 10.1002/ece3.3055

**Published:** 2017-05-30

**Authors:** Dilli P. Rijal, Torbjørn Alm, Lennart Nilsen, Inger G. Alsos

**Affiliations:** ^1^ Department of Natural SciencesTromsø MuseumUiT‐The Arctic University of NorwayTromsøNorway; ^2^ Department of Arctic and Marine BiologyUiT‐The Arctic University of NorwayTromsøNorway

**Keywords:** biodiversity, biological invasion, exotic species, invasibility, invasion ecology, structural equation model

## Abstract

The impact of invasion on diversity varies widely and remains elusive. Despite the considerable attempts to understand mechanisms of biological invasion, it is largely unknown whether some communities’ characteristics promote biological invasion, or whether some inherent characteristics of invaders enable them to invade other communities. Our aims were to assess the impact of one of the massive plant invaders of Scandinavia on vascular plant species diversity, disentangle attributes of invasible and noninvasible communities, and evaluate the relationship between invasibility and genetic diversity of a dominant invader. We studied 56 pairs of *Heracleum persicum* Desf. ex Fisch.‐invaded and noninvaded plots from 12 locations in northern Norway. There was lower native cover, evenness, taxonomic diversity, native biomass, and species richness in the invaded plots than in the noninvaded plots. The invaded plots had nearly two native species fewer than the noninvaded plots on average. Within the invaded plots, cover of *H. persicum* had a strong negative effect on the native cover, evenness, and native biomass, and a positive association with the height of the native plants. Plant communities containing only native species appeared more invasible than those that included exotic species, particularly *H. persicum*. Genetic diversity of *H. persicum* was positively correlated with invasibility but not with community diversity. The invasion of a plant community by *H. persicum* exerts consistent negative pressure on vascular plant diversity. The lack of positive correlation between impacts and genetic diversity of *H. persicum* indicates that even a small founder population may cause high impact. We highlight community stability or saturation as an important determinant of invasibility. While the invasion by *H. persicum* may decrease susceptibility of a plant community to further invasion, it severely reduces the abundance of native species and makes them more vulnerable to competitive exclusion.

## 1 INTRODUCTION

The impact of plant invasion is likely to increase in Europe. For instance, central European plant species from more productive areas have been predicted to become globally successful invaders (Dostál, Dawson, van Kleunen, Keser, & Fischer, 2013; Kalusová, Chytrý, Kartesz, Nishino, & Pyšek, 2013). Regardless of origin, the invasion success of a species depends on its capacity to colonize, survive, reproduce, and spread widely in a novel environment (Mitchell et al., 2006). However, exotic invasive species may reproduce and spread quicker than native invasive species (see Carey, Sanderson, Barnas, & Olden, 2012; Marrs et al., 2010; Muñoz‐Vallés & Cambrollé, 2015 for definition), as the former are resistant to strong disturbances and lack natural enemies in the introduced range (Keane & Crawley, 2002; Moles et al., 2012; Tilman, 2004). Due to such discrepancies in life‐history strategy between native and the exotic species, as well as complex interactions among biotic and abiotic factors, generalizing the impact of invasion and predicting the invasibility of a community remains challenging (Hulme, Pyšek, Pergl, Schaffner, & Vilà, 2014).

Meanwhile, recent progress in theoretical and experimental ecology makes it possible to estimate the impact of invasion based on the interplay between intrinsic characteristics of a community (Chytrý et al., 2008; Guo et al., 2015; Rejmánek, 2013). The degree of invasion is a measure of impact of exotic invaders on an invaded community (Guo et al., 2015). In particular, it indicates the level of dominance, constrained by biotic interactions, of exotic species in a community once they become invasive (Theoharides & Dukes, 2007; Williamson & Fitter, 1996). Thus, further growth and spread of an exotic species largely depends on the biotic interactions, especially competition for resources, within a community. Whether an exotic species can significantly impact a community also depends on the vegetative and reproductive capacity of that particular species (Gooden & French, 2015; Hejda, Pyšek, & Jarošík, 2009). The degree of invasion is likely to be higher in a community if the exotic invader is a superior resource competitor compared to resident species. In general, highly invasive exotic species maintain their dominance over native congeners across a wide range of environmental gradients, such as moisture and light, via continuous growth over the entire growing season (Čuda, Skálová, Janovský, & Pyšek, 2015). Thus, the cover difference between native and exotic species is the most important determinant of the impact of invasive species (Hejda et al., 2009; Pyšek & Pyšek, 1995).

Although there is no consensus on whether invasion leads to species extinction (Gurevitch & Padilla, 2004; Moles et al., 2012; Sax et al., 2007), it is generally assumed that the dominance of invasive exotics can affect population dynamics of native species over a longer period, and the persistence of such a phenomenon over the entire range of the native species may lead to its extinction (Lockwood, Hoopes, & Marchetti, 2013). At a very local scale, invasive exotics may reduce abundance of native species, which in turn may decrease species diversity of the invaded system (Hejda et al., 2009; Pyšek & Pyšek, 1995; Vilà et al., 2011). Thus, it should be noted that a native species should pass through low‐abundance stages with reduced distribution before it is extirpated (Wilsey & Potvin, 2000). Such small‐scale changes can be tracked by community characteristics such as abundance. Although native species richness is often negatively correlated with the abundance of exotic invasive species (Bernard‐Verdier & Hulme, 2015), there should be a noticeable decrease in the abundance of native species long before species richness starts declining (Mulder et al., 2004).

Invasibility, or the susceptibility of a community to biological invasion, is primarily an intrinsic characteristic of a community that reflects the number of vacant niches, which in turn is largely determined by available resources (Davis, Grime, & Thompson, 2000; Guo et al., 2015). It should be noted that if a community is already invaded by exotic species, total species (native and exotic) should be considered as “residents” while estimating future invasibility (Guo et al., 2015; Simberloff & Von Holle, 1999), as vulnerability of an ecosystem to invasion also depends on native–exotic and exotic–exotic interactions. For example, the first exotic invader may potentially increase or decrease the invasibility of an ecosystem (Catford, Vesk, Richardson, & Pyšek, 2012). The interplay between species composition, diversity, and biomass influences resource availability in a particular community (Catford et al., 2012), which in turn determines whether a community favors “establishment” of exotic invaders (Theoharides & Dukes, 2007; Williamson & Fitter, 1996).

Species‐rich habitats are less prone to novel invasion than species‐poor habitats; that is, biodiversity acts as a barrier to biological invasion (Kennedy et al., 2002); and the loss of species may decrease the invasion resistance of a community at local scales, that is, neighborhood scales (Levine, 2000). Evenness is considered as an indicator of a community's resistance to biological invasion (Shochat & Ovadia, 2011). Similarly, the negative relationship between biomass and invasibility (Jiang, Zhang, & Wang, 2007) indicates that a community may resist biological invasion if the native species are highly abundant or produce huge biomass (Guo, 2015). Thus, evenness, total richness, and biomass of a community can be considered indicators of community saturation that reflects whether a community is resistant or vulnerable to biological invasion.

The genetic diversity of a particular invader has been reported to have positive association with invasion success (Crawford & Whitney, 2010). However, negative (Crutsinger, Souza, & Sanders, 2008) and neutral relationships between genetic diversity and invasibility (Hovick, Gümüşer, & Whitney, 2012; Vellend, Drummond, & Tomimatsu, 2010; Weltzin, Muth, Von Holle, & Cole, 2003) are also common. While a few studies have reported how genetic diversity of native species influences the establishment success of exotic species (e.g., De Meester, Louette, Duvivier, Van Damme, & Michels, 2007), whether genetic diversity of a dominant exotic invader shapes the future of invasion dynamics is rarely emphasized. In some cases, invasion history shapes the genetic diversity pattern of the exotic invader (e.g., Rijal, Alm, Jahodová, Stenøien, & Alsos, 2015). It therefore remains unclear whether invasion history and residence time of dominant invaders, or genetic diversity per se, shapes invasion dynamics.

*Heracleum persicum*, a herbaceous perennial invasive plant native to Iran, was introduced to Europe via England (Rijal, Alm, et al., 2015). It is more likely to invade suitable habitats of the rest of continental Europe as it is already well established in Scandinavia. The species has been blacklisted in Norway (Gederaas, Moen, Skjelseth, & Larsen, 2012) and included in the invasive alien species list of EU concern (Council Regulation, 2016). It has been recommended as a quarantine pest in the European and Mediterranean region (EPPO 2009) due to its rapid spread, extensive growth, and the negative effect it may have on biodiversity. However, community characteristics, if any, that favor the invasion of *H. persicum* remain largely unexplored. In addition, how *H. persicum* affects biodiversity at the community level, using species richness and diversity indices, awaits further quantification. Thus, our aims were to (1) assess the impact of *H. persicum* on native plant abundance and diversity, (2) estimate invasibility of plant communities based on the current level of invasion, and (3) evaluate the relationship between genetic diversity of *H. persicum* and invasibility.

## 2 MATERIALS AND METHODS

### 2.1 Vegetation sampling

**Figure 1:**
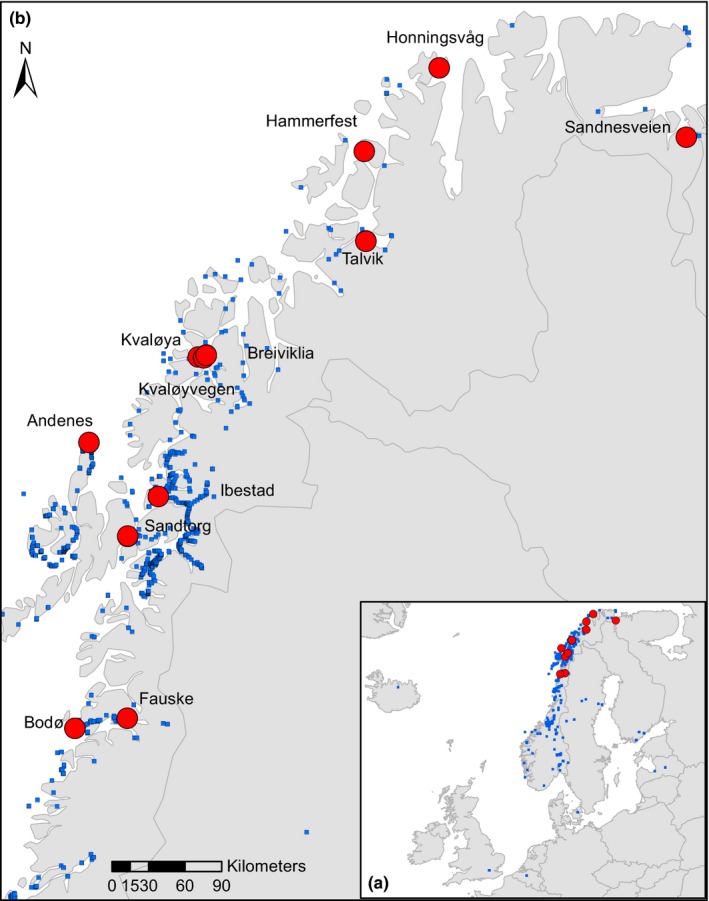
An enlarged map of the current sampling locations (large circles, a), and historical records (small squares) of *Heracleum persicum* in the introduced range (b)

In its introduced range, *H. persicum* is widespread in central and northern Norway (Alm, 2013). It is also found at scattered stations in southern Norway as well as in Denmark, England, Finland, Iceland, Latvia, and Sweden (Fröberg, 2010; Rijal, Alm, et al., 2015; also see Figure 1). *Heracleum persicum* predominantly occupies human‐disturbed sites, including abandoned agricultural land, or sites close to the sea which appear nutrient‐rich, perhaps due to the influence of sea algae and other organic compounds. Geographically, the sampling area encompassed middle boreal, northern boreal, and low alpine vegetation zones of Norway (as defined by Fremstad, 1998). Based on species composition, Fremstad (1998) has described 24 major groups of vegetation that have been further classified into 137 vegetation types and 379 subtypes in Norway. *Heracleum persicum* mostly occurs in different vegetation types of anthropogenically disturbed sites (Fremstad, 1998), such as urban thermophilous weed vegetation, vegetation on road embankments and waste places, vegetation on trampled ground, weed vegetation in cultivated fields, and strongly fertilized vegetation. Some of the dominant species of those vegetation types were *Achillea millefolium, Alchemilla subcrenata, Anthriscus sylvestris, Cirsium arvense, Deschampsia cespitosa, Epilobium angustifolium, Equisetum arvense, Festuca rubra, Filipendula ulmaria, Galeopsis tetrahit, Geranium sylvaticum, Poa trivialis, Ranunculus acris, Senecio vulgaris, Taraxacum officinale, Tussilago farfara*, and *Urtica dioica*.

Vegetation was sampled during July–August 2012 and August 2013 within the area where *H. persicum* was most frequent (Figure 1). The sampling approach was to compare species richness and diversity estimates between *H. persicum*‐invaded and noninvaded plots. We sampled five invaded and five noninvaded plots in each location except Bjørnevatn, for which only two plots were sampled. The noninvaded plots were established as close as possible to the invaded plots to minimize variation in site conditions and to insure that the noninvaded plots represented vegetation before the invasion of *H. persicum* (space‐for‐time substitution approach; Pickett, 1989; Pyšek & Pyšek, 1995). We selected a homogenous stand of *H. persicum* wherever possible and covered variation in the growth form of *H. persicum* while sampling. The geographic coordinates of each location are provided in Table 1. The percent cover of each species within 2 × 2 m^2^ plots was visually estimated from 56 invaded and noninvaded plot pairs. Resident species formed different vegetation layers, and as a consequence, total cover exceeded 100% in most of the plots. Elven (2005) was followed for nomenclature. Herbarium vouchers of *H. persicum* and some other taxa collected for identification have been deposited at Tromsø Museum (TROM).

**Table 1:** Sampling locations of *Heracleum persicum* in northern Norway. Average expected heterozygosity (Nei's genetic diversity) represents bootstrapped mean of original values after 10,000 replications (see Section 2)

County	Municipality	Location	Latitude	Longitude	Altitude	Genetic diversity
Finnmark	Hammerfest	Elvetun	70.6656	23.6985	17	0.192
Finnmark	Honningsvåg	Elvebakken	70.9945	25.9732	11	0.186
Finnmark	Sør‐Varanger^a^	Bjørnevatn	69.6754	29.9626	46	NA
Finnmark	Alta	Talvik	70.0472	22.9631	37	0.232
Nordland	Andøy	Andenes	69.3222	16.1259	17	0.126
Nordland	Tjeldsund	Sandtorg	68.5675	16.3502	9	0.140
Nordland	Fauske	Sjøgata	67.258	15.3847	6	0.196
Nordland	Bodø	Plassen	67.2865	14.396	11	0.136
Troms	Tromsø	Kvaløya	69.6836	18.808	10	0.188
Troms	Tromsø	Kvaløyvegen	69.6662	18.9107	1	0.172
Troms	Tromsø	Breiviklia	69.6785	18.977	22	0.262
Troms	Ibestad^b^	Ibestad	68.7868	17.1563	8	0.164

NA, not available.

a Only one pair of invaded/noninvaded plots sampled.

b Sampled in August 2013.

### 2.2 Genetic diversity

Data on genetic diversity were used from Rijal, Alm, et al. (2015). These data include 575 individuals of *H. persicum* screened for 25 microsatellite markers following the methods in Rijal, Falahati‐Anbaran, Alm, and Alsos (2015).

### 2.3 Data analysis

Species were classified into native and exotic based on the origin of species following Gederaas et al. (2012). All the variables reported in this study are defined in Box 1 or this section. Diversity was estimated as species richness, evenness, and taxonomic diversity. All the species present in a plot were counted as total species richness, which was further divided into native richness and exotic richness. All the species were further classified into grasses and herbs after removing the few woody species, and native grass and herb richness was calculated. Presence/absence of *H. persicum* was the primary factor to discriminate invaded and noninvaded plots. Thus, *H. persicum* was excluded when calculating species richness (Hejda & Pyšek, 2006). However, it was included during the estimation of evenness and taxonomic diversity, as the total abundance of species is the major determinant of such estimations (Thomsen, Wernberg, South, & Schiel, 2016). The covers of species were included as importance values while calculating Pielou's evenness (J), as suggested by Hill (1973). The taxonomic diversity (∆), a measure of average taxonomic distance between two species, was calculated following Clarke and Warwick (1998). Cover of *H. persicum* was considered as an indicator of invasion success. Height variances of total and native species were calculated, as well as height and cover differences between 10 dominant native species (Table S1) and *H. persicum*.

Box 1 **Definitions of variables. Each variable was estimated per plot, unless otherwise stated**

1

**Table:** 

Variables	Definition
Dominants’ cover difference	Cover difference between *Heracleum persicum* and ten dominant species.
Dominants’ height difference	Height difference between *Heracleum persicum* and ten dominant species.
Exotic cover	Total cover of exotic species.
Exotic richness	Total count of exotic species.
Genetic diversity	Nei's genetic diversity estimated using microsatellite markers for *Heracleum persicum* in a location.
Maximum biomass	The highest total biomass recorded among ten invaded/noninvaded plots in a location.
Maximum richness	The highest number of species recorded among ten invaded/noninvaded plots in a location.
Native biomass	Biomass estimated using native cover and native height.
Native cover	Total cover of native species.
Native height	Average height of native species.
Native height variance	Variance in the native height from the mean height of native species.
Native richness	Total count of native species.
Nitrogen	Average nitrogen estimated from Ellenberg's indicator values.
Relative exotic richness	Proportion of exotic richness.
Total/observed biomass	Total biomass estimated using total cover and total height.
Total cover	Total cover of species.
Total height	Average height of all species.
Total height variance	Variance in the total height from the mean height of all species.
Total/observed species richness	Total count of species.

Ellenberg's indicator values (EIVs) for light (L), moisture (F), and nitrogen (N) were assigned to each species if available (Ellenberg et al., 1992). We performed a modified randomization test to select the most important EIV (Zelený & Schaffers, 2012). Only nitrogen remained significant after the modified randomization test and was retained for further analyses. To avoid labor‐intensive destructive sampling for biomass estimation, we used cover from visual estimation and mean height of each species from the standard Norwegian flora (Elven, 2005) while estimating biomass. The biomass volume was calculated by adjusting for herb layer overestimation (100% herb layer in our case as there was no exposed soil in sampled plots) as suggested by Axmanová et al. (2012). *Heracleum persicum* was included while estimating total biomass.

To evaluate the change in biomass of the native species as a consequence of *H. persicum* invasion, we generalized the equation of Hulvey & Zavaleta, 2012);. Change in the native biomass per unit change of the invader biomass was considered as the population‐level impact of *H. persicum* invasion, and the impact metric is 0 when biomass of the native species is unaffected by the invasion of *H. persicum*; −1 when each unit increase in invader biomass displaces the same unit of the native biomass; and 1 if each unit increase in invader biomass increases the same unit of the native biomass (Hulvey & Zavaleta, 2012). We used a one‐sample *t* test to evaluate whether there was a significant impact of *H. persicum* invasion on native biomass. Relative proportion of exotic richness and abundance were also calculated to evaluate the impact of *H. persicum* invasion (sensu Catford et al., 2012). Impact of invasive species may vary among invaded sites based on the invasion histories, which in turn also shapes invasion dynamics (Guo et al., 2015). Thus, to evaluate susceptibility of different sites to further invasion, invasibility (*I*) was calculated as follows (p. 2,618, equation 2, Guo et al., 2015):

$$\text{Invasibility}\;\left(I\right)=1-\left(S_{\mathrm{obs}}/S_{\max}+B_{\mathrm{obs}}/B_{\max}\right)/2$$

where *S*_obs_ and *S*_max_ are observed and maximum richness and *B*_obs_ and *B*_max_ are observed and maximum biomass in a community, respectively.

Given that species richness and biomass maxima of each location may vary, local species richness and biomass maxima were used for each location when calculating invasibility. The invasibility was estimated for each plot, and the average value of the paired invaded and noninvaded plots was used in further analyses. We used nonparametric analyses when data did not meet the assumptions of parametric tests. A Kruskal–Wallis rank‐sum test was used to compare the invasibility and impact on biomass among different sites, and Dunn's test was used for multiple comparisons. We used Bonferroni's method for *p*‐value adjustment. We considered location as the block and performed split‐plot analysis considering plots as nested within locations (Crawley, 2013). We used linear mixed‐effects models (“lme4 package”; Bates, Maechler, Bolker, & Walker, 2015) accounting for random error of locations while comparing diversity estimates and other environmental variables of the invaded and noninvaded plots. *p*‐values were obtained by likelihood ratio tests of the full model, with plot type as a fixed effect against the null model without the fixed effect. Pearson's product–moment correlation was used to assess correlation among variables, and only one biologically meaningful variable was selected for further analysis.

To assess the relationship between invasibility and genetic diversity, average invasibility of each site, except Bjørnevatn, was regressed against average genetic diversity of *H. persicum*. We resampled genetic diversity and invasibility 10,000 times for each location in a group of five samples with replacement. Bootstrapped means of genetic diversity (Table 1) and invasibility for each location were used in regression analysis. A bootstrapped *R*‐squared value was calculated after 10,000 replications. Ordinary least squares regression was used with linear and quadratic terms, and only significant terms were retained. Sørensen's index of dissimilarity was used to compare beta diversity between the invaded and noninvaded plots based on the presence/absence data. To evaluate how dominant invader and native species influence community invasibility, a path analysis was performed using structural equation modeling (SEM) (Bollen, 1989) as it is one of the suitable methods for studying hypotheses about multiple processes operating in systems (Grace, Anderson, Olff, & Scheiner, 2010). In our first model, we hypothesized that cover of dominant native species can directly affect invasibility and influence the abundance of an exotic invader (*H. persicum*) which in turn mediates community invasibility. The dominant's cover was replaced by cover of all native species in the second model. The analysis was performed using R package lavaan (Rosseel, 2012). All the analyses were performed in R version 3.3.1 (R Core Team 2016).

## 3 RESULTS

### 3.1 Overall vegetation composition

A total of 90 species, including *H. persicum*, were recorded from the study area (Table S1). The invaded and noninvaded plots contained 67 and 77 species, of which 13 and 23 species were unique to invaded and noninvaded plots, respectively. In total, 54 species were shared by both. The species number was reduced by about 13% in the invaded compared to the noninvaded plots. A total of seven exotic species, including *H. persicum*, were found within the study area. *Epilobium adenocaulon*,* Lilium martagon*, and *Primula elatior* occurred in the invaded plots, whereas *Aquilegia vulgaris*,* Lysimachia punctata*, and *Ribes uva‐crispa* occurred only in the noninvaded plots. *Epilobium adenocaulon* occurred in three plots. The other non‐native species, except *H. persicum*, occurred only once with a cover range of 1%–3% for five species and 20% for *Ribes uva‐crispa*. Five different families were represented with a single non‐native species, and only Primulaceae was represented twice.

Species such as *Anthriscus sylvestris*,* Epilobium angustifolium*, and *Equisetum arvense* tended to occur frequently in the invaded plots, whereas *Taraxacum officinale*,* Geranium sylvaticum*, and *Achillea millefolium* were more frequent and dominant in the noninvaded plots (Table S1). In general, invaded and noninvaded plots had similar beta diversity as reflected by Sørensen's index of dissimilarity (0.74 and 0.75, *p *= .186).

### 3.2 Impact of *Heracleum persicum* on plant diversity and abundance

**Figure 2:**
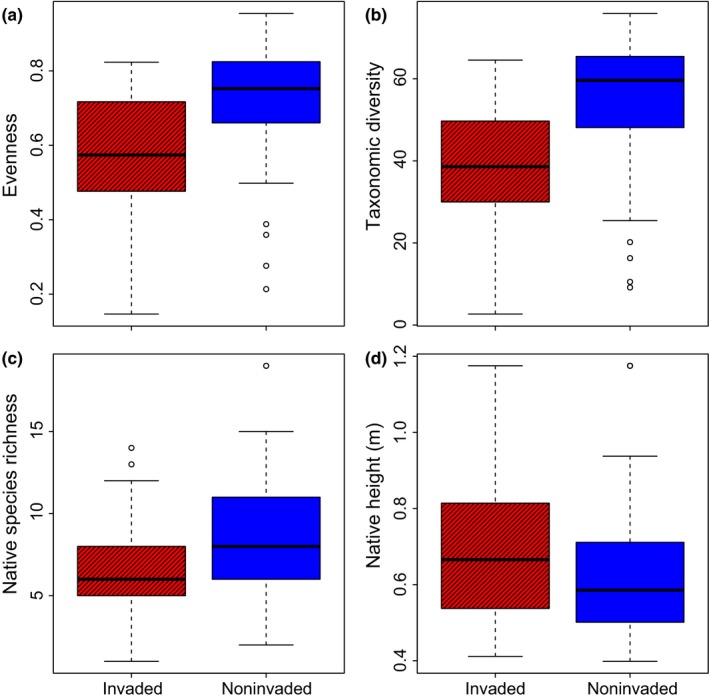
Box plots showing differences in (a) evenness, (b) taxonomic diversity, (c) natives’ species richness, and (d) natives’ plant height between the invaded (with pattern) and the noninvaded (without pattern) plots across locations. The test statistics of linear mixed‐effects models with invaded/noninvaded plots nested within locations are provided in Table 2. *Heracleum persicum* was excluded from species richness calculation

We observed significantly lower native cover, evenness, taxonomic diversity, native richness, native grass richness, and herb richness in the invaded compared to the noninvaded plots (Figure 2a–c, Table 2; Fig. S1b–d). On average, the invaded plots contained two native species fewer than noninvaded plots (Table 2). In contrast, native plant height was higher in the invaded than in the noninvaded plots (Figure 2d). Of the 54 common native species, 47 species had lower cover and seven species had higher cover in the invaded compared to the noninvaded plots (Table S1). The impact of invasion was higher on native grass richness compared to native herb richness. The invasion reduced 35% of the grasses and 18% of the herb from the invaded plots compared to noninvaded plots (Table 2, Fig. S1c,d).

**Table 2:** Results of linear mixed‐effects models with invaded/noninvaded plots as fixed effect and locations as random effect for different response variables with 107 degrees of freedom. *p*‐values < .05 are in the bold face and were generated for maximum likelihood tests between models with and without fixed effect (null model). Random effect was present in all the models. Mean values of response variables are provided for the invaded plots; however, values provided for the noninvaded plots indicate the difference from the invaded plots. Heracleum persicum was not included in species richness calculation

Response variables	Model component	Estimates	*SE*	*t*‐Value	*SD*	*p*‐Value
Evenness	Fixed effect					
	Invaded	0.582	0.027	22.827		**.000**
	Noninvaded	0.141	0.027	5.299		
	Random effect					
	Plot:Location				0.000	
	Location				0.058	
	Residual				0.141	
Taxonomic diversity	Fixed effect					
	Invaded	39.096	2.563	15.257		**.000**
	Noninvaded	15.472	3.456	4.477		
	Random effect					
	Plot:Location				5.470	
	Location				2.621	
	Residual				13.717	
Native richness	Fixed effect					
	Invaded	6.643	0.622	10.679		**.001**
	Noninvaded	2.000	0.520	3.846		
	Random effect					
	Plot:Location				0.000	
	Location				1.712	
	Residual				2.752	
Native height	Fixed effect					
	Invaded	0.705	0.041	17.157		**.031**
	Noninvaded	−0.063	0.026	−2.396		
	Random effect					
	Plot:Location				0.026	
	Location				0.126	
	Residual				0.127	
Native cover	Fixed effect					
	Invaded	56.646	5.090	11.130		**.000**
	Noninvaded	84.026	5.675	14.800		
	Random effect					
	Plot:Location				5.156	
	Location				10.634	
	Residual				27.769	
Native biomass	Fixed effect					
	Invaded	25.885	3.819	6.779		**.000**
	Noninvaded	42.785	4.878	8.772		
	Random effect					
	Plot:Location				9.633	
	Location				5.617	
	Residual				14.713	
Native grass richness	Fixed effect					
	Invaded	1.312	0.203	6.464		**.002**
	Noninvaded	0.714	0.189	3.782		
	Random effect					
	Plot:Location				0.000	
	Location				0.520	
	Residual				0.999	
Native forb richness	Fixed effect					
	Invaded	5.276	0.497	10.607		**.005**
	Noninvaded	1.161	0.400	2.902		
	Random effect					
	Plot:Location				0.000	
	Location				1.397	
	Residual				2.116	
Average nitrogen	Fixed effect					
	Invaded	6.262	0.185	33.840		.196
	Noninvaded	−0.200	0.154	−1.300		
	Random effect					
	Plot:Location				0.000	
	Location				0.510	
	Residual				0.816	
Invasibility	Fixed effect					
	Invaded	0.264	0.023	11.540		**.000**
	Noninvaded	0.229	0.018	12.760		
	Random effect					
	Plot:Location				0.000	
	Location				0.065	
	Residual				0.095	

**Figure 3:**
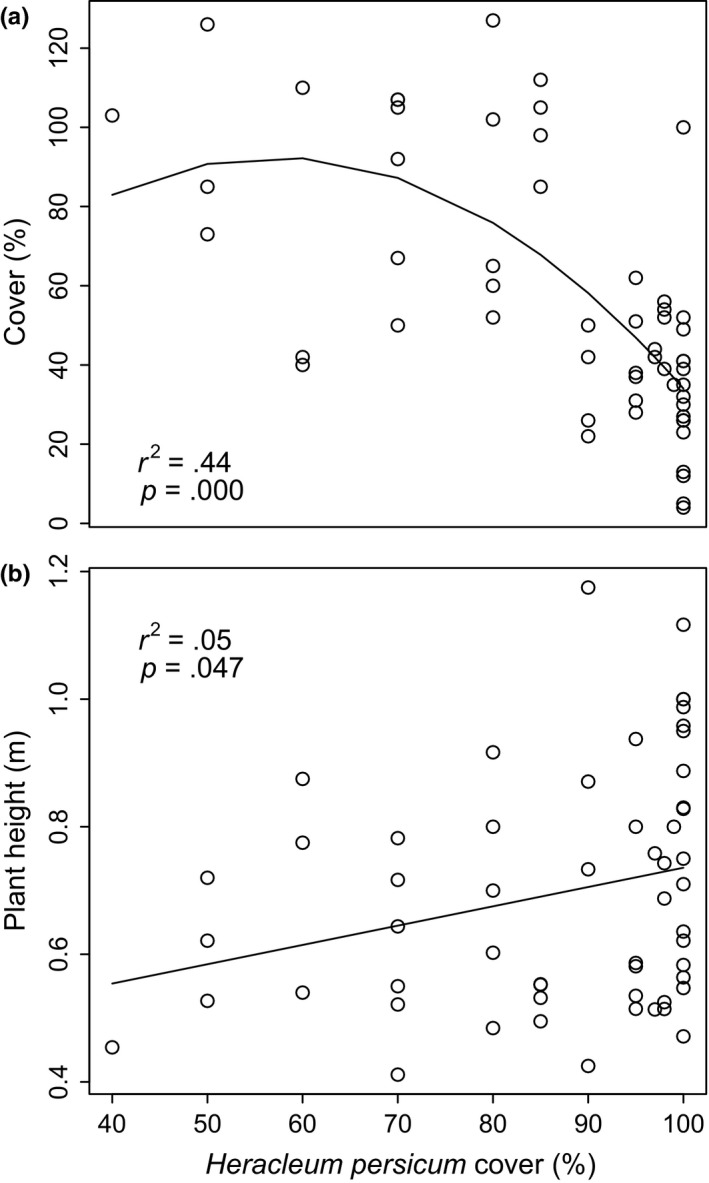
Impact of cover of *Heracleum persicum* on (a) natives’ cover and (b) natives’ height

*Heracleum persicum* cover had a negative nonlinear relationship with native cover (Figure 3a and Table S2). Taller native plants co‐occurred with *H. persicum*, as indicated by the positive correlation between native plant height and *H. persicum* cover (Figure 3b).

**Figure 4:**
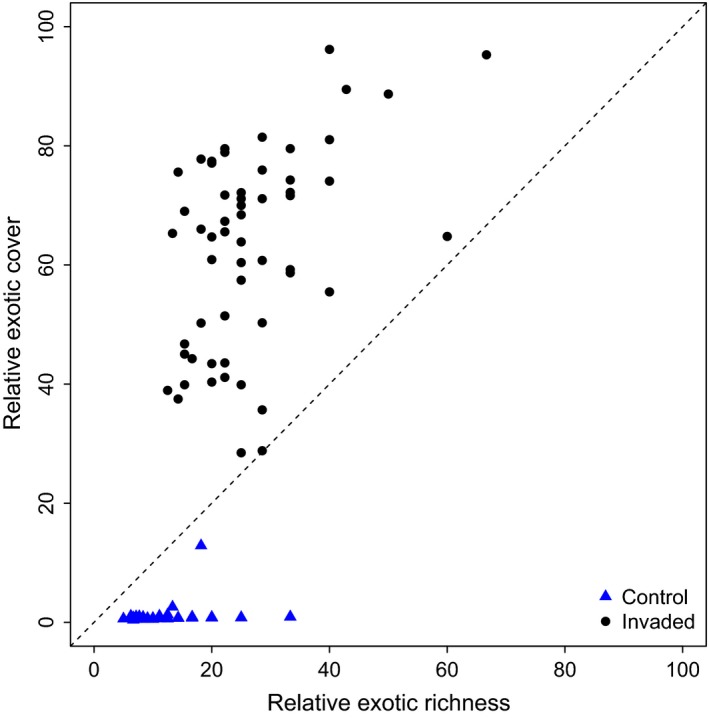
Relative exotic richness and cover in the invaded (circle) and the noninvaded (triangle) plots. Despite lower exotic richness both in the invaded and the noninvaded plots, there is a high relative cover of exotic species in the invaded plots particularly due to the high abundance of *Heracleum persicum*

Exotic richness was low in both the invaded and noninvaded plots. Relative exotic abundance was also lower for the noninvaded plots. However, the invaded plots had extremely high relative exotic abundance, consisting primarily of *H. persicum* (Figure 4).

**Figure 5:**
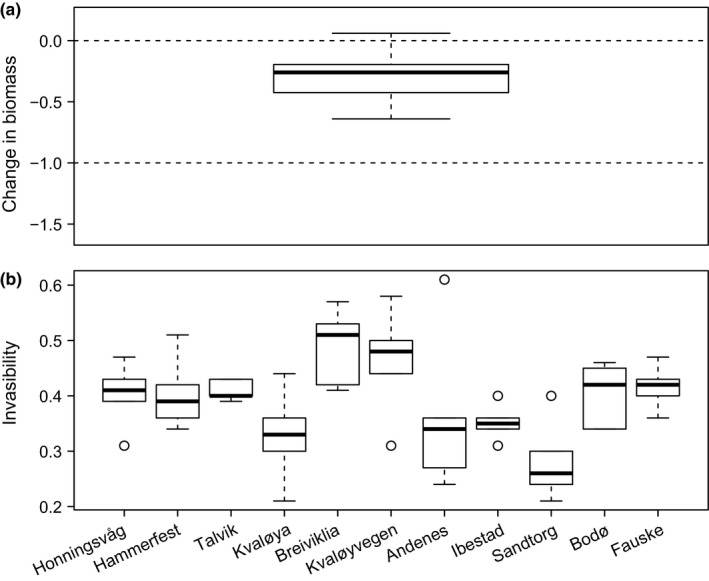
Impact of *Heracleum persicum* in the invaded sites as reflected by (a) the change in natives’ biomass across locations (X‐axis noninformative) and (b) estimated invasibility for different locations. Sites are ordered by decreasing latitude from the left to the right

There was a significant negative impact of *H. persicum* invasion on native's biomass (*t*_54_ = −15.04, *p *= .000; Figure 5a). The impact of *H. persicum* on native biomass appeared higher in Sandtorg, Bodø, and Breiviklia than in other sites as indicated by the loss of biomass of native species (Table S3). Andenes appeared as the least affected site. However, a marginally nonsignificant Kruskal–Wallis rank‐sum test (χ^2^_10_ = 18.17, *p *= .052) and Dunn's test (Table S3) revealed that the difference of invasion impact was not significant among sites.

### 3.3 Invasibility and genetic diversity

**Figure 6:**
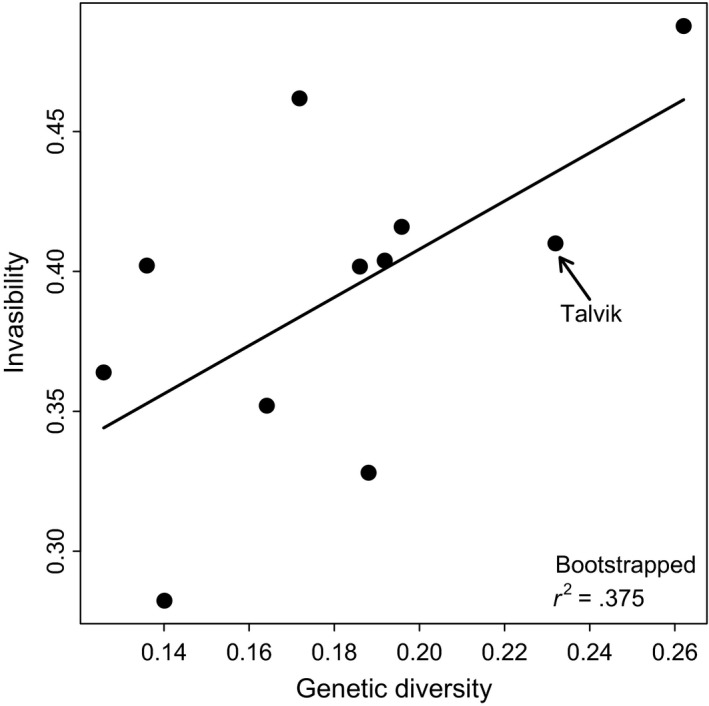
Relationship between average genetic diversity of *Heracleum persicum* and invasibility for 11 sites from northern Norway. Talvik represents approximate point of the first introduction of *H. persicum* in northern Norway. A single sample from Bjørnevatn was not included in the regression. *R*‐squared value was generated after 10,000 bootstrapping

**Figure 7:**
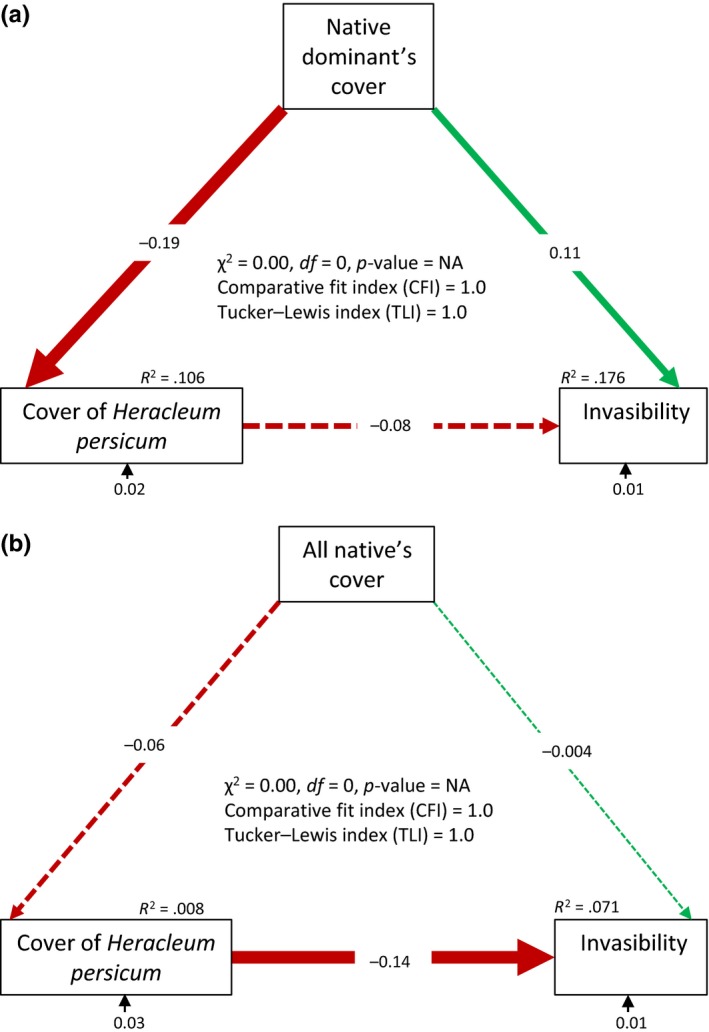
Structural equation model representing relationship of *Heracleum persicum* cover and invasibility with cover of (a) 10 dominant native species and (b) all the native species. Zero χ^2^ score and degree of freedom, and comparative fit index (or Tucker–Lewis index) of 1 indicate tight model‐data fit. Solid and broken arrows indicate significant and nonsignificant relationships, respectively. The values on the arrows are standardized path coefficients indicating associations (− indicates negative association). The *R* ^2^ values are provided for response variables

The estimated invasibility was significantly different in at least one site as indicated by a Kruskal–Wallis test (χ^2^_10_ = 22.29, *p* = .014). The highest invasibility was estimated for Breiviklia followed by Kvaløyvegen (Figure 5b and Table S4). The lowest invasibility was estimated for Sandtorg followed by Andenes. However, after *p*‐value adjustment, post hoc Dunn's test indicated only Sandtorg as significantly differentiated from Breiviklia (Table S4). Invasibility was negatively correlated with *H. persicum* cover, total height, total height variance, and cover difference of dominant native species with *H. persicum*. It was, however, positively correlated with native cover and biomass, evenness, taxonomic diversity, and genetic diversity of *H. persicum* (Figure 6 and Table S2). There was a significant positive association between cover of dominant native species and invasibility. However, it had a significant negative impact on *H. persicum* cover, which in turn had a nonsignificant negative effect on invasibility (Figure 7a). On the other hand, cover of overall native species had no significant direct impact on cover of *H. persicum* and invasibility. Meanwhile, it influenced the effect of cover of *H. persicum* on invasibility leading to a significant negative impact (Figure 7b).

## 4 DISCUSSION

### 4.1 Impact of *Heracleum persicum* on plant diversity and abundance

Invasion of *H. persicum* consistently reduced all the diversity parameters in the invaded plots compared to the noninvaded plots. Previous studies have reported a similar pattern for *Heracleum mantegazzianum* (Hejda et al., 2009; Pyšek & Pyšek, 1995), particularly when invaders had larger size and higher cover than native species (Hejda et al., 2009). The apparent pattern may be the result of higher cover of *H. persicum* than other native species, as cover of exotic invasive species is considered one of the most important factors in reducing native diversity and evenness (Hejda et al., 2009). We found lower relative exotic richness than relative exotic abundance, especially of *H. persicum*. Such a pattern may indicate the presence of a single dominant exotic invader that can reduce diversity and potentially extirpate native species (Catford et al., 2012). In our case, high relative exotic abundance in the invaded plots indicates monopolization by *H. persicum,* which in turn poses a risk of competitive exclusion of native species. *Heracleum persicum* attains a height of up to 3 m and produces huge biomass and extensive cover (Nielsen, Ravn, Nentwig, & Wade, 2005). It grows rapidly in late spring and early summer and thereby minimizes competition with the resident vegetation. In addition, due to high stature and dense cover, it may shade other resident species. As a consequence, resident vegetation may not get enough resources for germination and growth, which may reduce species richness in the invaded plots. In addition, *H. persicum* may inhibit the germination of seeds of other species due to its allelopathic effect (Myrås & Junttila, 1981). Of the two distinct plant invasion strategies identified, that is, exploiters versus coexisters (Lai, Mayfield, Gay‐des‐combes, Spiegelberger, & Dwyer, 2015), *H. persicum* appears as an efficient “exploiter” that may reduce native species richness due to the possession of competitive traits (e.g., early growth, huge biomass production, enormous seed production, and perennial habit).

If we assume that the vegetation composition of invaded and noninvaded plots was similar before the invasion (see Hejda & Pyšek, 2006; Nielsen, Whigham, Frew, Callaway, & Dickinson, 2015 for a similar study with same assumption), then plant diversity was significantly decreased in the invaded plots. In general, impact of invasion should be visible in terms of reduced abundance of native species, which is the case here, and which may ultimately cause local extirpation leading to reduced native richness (Mulder et al., 2004; Wilsey & Potvin, 2000). The evenness was positively correlated with native cover and richness and negatively correlated with *H. persicum* cover. High evenness is assumed when there is greater height variation among the plant species (Wilsey & Potvin, 2000). The evenness may also depend on the difference in the cover and height of the invader and dominant native species (Hejda et al., 2009). In our case, the height difference of dominant native species had a positive effect on evenness; however, evenness had a negative relationship with the cover difference between *H. persicum* and dominant native species (Table S2). High interspecific competition for light is predicted when there is a difference in plant architecture among species (Wilsey & Potvin, 2000). It looks like *H. persicum* reduced the cover of other native species due to either high competition or shading, but at the same time allowed a limited number of species to co‐occur. Probably few taller native species, which can compete with *H. persicum* for light, have co‐occurred with it. The diversity indices are based on the number and abundance of species, and accordingly, we observed a negative impact of reduced abundance of native species on all the diversity estimates. Thus, the reduced cover of most native species in the invaded plots indicates that several species are on their way to local extinction if *H. persicum* continually exerts such pressure on them.

Overall, the invasion of *H. persicum* had a negative impact on Norwegian vascular plant diversity. A high negative impact of exotic invader is more likely to be detected at the local scale, due to either biotic interactions or sampling bias (Carboni et al., 2013; Fridley et al., 2007). One may claim our result is a consequence of a statistical artifact as a large exotic invader tends to occupy most of the smaller plot leaving fewer chances for native species to be sampled (sensu Fridley et al., 2007). However, we argue that cover of *H. persicum*, which ranged from 40% to 100% per plot, had no significant correlation with the native species richness within the invaded plots (Table S1) indicating an absence of plant‐size‐dependent sampling bias. We also emphasize that the selection of sample size depends on the objectives of a study. Our aims were to study the impact of *H. persicum* invasion at plot level, that is, biotic interactions at local scale (Carboni et al., 2013; Fridley et al., 2007). Due to homogenous stands of *H. persicum*, it was difficult to find large patches of noninvaded area in the vicinity of an invaded area sharing a similar history and environment. In such a case, larger plots would have forced us to study more heterogeneous noninvaded plots compared to invaded plots making it difficult to disentangle whether the observed differences were due to invasion or habitat heterogeneity. Thus, the selected sample size was appropriate for addressing our aims, particularly the postinvasion impact of an exotic invader at a local scale.

### 4.2 Overall exotic species composition

In contrast to the synergistic effect or invasional meltdown hypothesis that emphasizes the positive role of exotic species in facilitating establishment and spread of other exotic species (Ricciardi, Hoopes, Marchetti, & Lockwood, 2013; Simberloff & Von Holle, 1999), our results indicate that *H. persicum* does not facilitate establishment of other exotic species. By reporting a high proportion of exotic invasive species co‐occurring with particularly dominant invasive species, several studies have supported the idea that exotic species facilitate each other's establishment, spread, and impact (e.g., French & Watts, 2015; Nielsen et al., 2015). However, our result does not support such an idea, as the other exotic species we found were overall few and scattered, and there was no difference in their occurrence between *Heracleum* invaded and noninvaded plots. The harsh climate of northern Norway, with a short summer and long winter, is a likely explanation for the general paucity of exotic species in our subarctic plots. Despite this, a large and rapidly increasing number of exotics have been recorded during recent years, for example, in Troms (Alm & Pedersen, 2015). This discrepancy may be the result of temporal variation in introduction of different species and their differential lag phases (Daehler, 2009; Kowarik, 1995; Larkin, 2012). In (northern) Norway, *H. persicum* was introduced at an earlier date than most other exotic species currently blacklisted (Gederaas et al., 2012), and has thus become invasive before the later introductions.

### 4.3 Determinants of invasibility

#### 4.3.1 Genetic diversity or invasion history?

The positive association between genetic diversity of *H. persicum* and invasibility contradicts the prevailing expectation that genetic diversity should have a negative impact on invasibility, as it is considered analogous to species diversity (Vellend & Geber, 2005). However, neither Elton's diversity–resistance hypothesis (Elton, 1958) nor the species richness–genetic diversity relationship (Vellend, 2005) is firmly established (Levine, Adler, & Yelenik, 2004; Taberlet et al., 2012). We emphasize that the introduction history of *H. persicum* in Norway is more important in determining invasibility than genetic diversity per se. The positive association between latitude and genetic diversity appears to be a consequence of the subsequent loss of genetic diversity during the north–south spread, most likely from an area close to Talvik, of *H. persicum* in Norway (Rijal, Alm, et al., 2015). This means genetically diverse northern populations of *H. persicum* had a longer residence time than those recently established populations in more southern Norway. As a result, due to succession, competitively strong native species co‐occur with *H. persicum* that may constrain its cover. The path analysis also indicated that cover of *H. persicum* is constrained by dominant native species (Figure 7a). It also revealed that *H. persicum* resists further invasion even if its abundance is not controlled by native species (Figure 7b). If so, older sites offer less competition, but high resources, to newcomers due to a low cover of the dominant invader compared to those where *H. persicum* is dominant. Alternatively, due to the gradual decrease in nitrogen with increasing latitude (Table S2), cover of nutrient‐demanding *H. persicum* is reduced leaving space for further invasion.

#### 4.3.2 Characteristics of an invaded community

We found a negative association (*R* = −0.46; see Table S2) between invasibility and *H. persicum* cover. Our finding agrees with the general conclusion that the presence of several exotic species indicates habitat heterogeneity and community saturation, which in turn makes the invaded community less prone to further invasion (Catford et al., 2012; Chytrý et al., 2012). The estimated invasibility was lower for areas with a high level of current invasion (e.g., Sandtorg; Figure 5b) and higher for areas with a low or average level of current invasion. Thus, as previously predicted for northwestern and northern Europe (Chytrý et al., 2012), our results also support the idea that areas with a low or average level of invasion may likely become more invasible in the future.

Our results indicated that noninvaded plots are relatively more susceptible to further invasion compared to invaded plots, as reflected by the higher estimated invasibility for noninvaded plots (Table 2). Similarly, we observed a positive association of invasibility with the native cover, as well as all other variables (native biomass, evenness, taxonomic diversity) where native species were the major contributors (Table S2). Such a result may indicate that the noninvaded community is unsaturated in terms of number of species and biomass and thus may be prone to further invasion (Case, 1990; Kennedy et al., 2002; Levine & D'Antonio, 1999; Oakley & Knox, 2013; Rejmánek, 1996). In contrast, invasibility had a negative association with total height, total height variance, and cover difference between 10 dominant native species and *H. persicum*. It is important to note the inclusion of exotic species in those calculations. Thus, our results suggest that the presence of a dominant exotic species or high degree of invasion (Guo et al., 2015) may provide resistance to further invasion. A great deal of trait variation is expected among coexisting species, and plant height is one of the most important traits determining coexistence among species (Falster & Westoby, 2005; Moles et al., 2009; Soliveres et al., 2014). In our case, the negative association between invasibility and total height variance intuitively suggests that many shade‐tolerant species of varying size may co‐occur with the giant *H. persicum,* which ultimately occupy the available space and deplete resources making further invasion unlikely. In other words, co‐occurrence of exotic and native species of varying size may fill the vacant niches and use most of the resources, thereby preventing further invasion (Eisenhauer, Schulz, Scheu, & Jousset, 2013; John & Jarrett, 2006). We highlight the fact that a high level of invasion also means an increased probability of the occurrence of exotic species leading to a higher risk of establishment and invasion by other exotic species (Chytrý et al., 2012; Rejmánek & Randall, 2004). Our data are not robust enough to test whether exotic species increase or decrease further invasion.

*Anthriscus sylvestris*,* A. subcrenata*, and *T. officinale* were among the most frequent species in the study area (Table S1). Elsewhere in Europe, *A. sylvestris* and *G. tetrahit*, which generally grow in open and disturbed habitats, have been considered as the indicator of potential sites more susceptible to invasion by non‐native species (Godefroid & Koedam, 2003). *Anthriscus sylvestris* is rapidly expanding in Norway, mostly in abandoned fields and other disturbed habitats. Our sampling strategy was not designed to disentangle whether the presence of *A. sylvestris* indicates habitats that may be invaded by *H. persicum*. However, the frequent occurrence of *A. sylvestris* in the invaded plots indicates human disturbance. In addition, the genetic diversity pattern of *H. persicum* within Norway (Rijal, Alm, et al., 2015) suggests that the long‐distance dispersal is rare and necessitates anthropogenic aid. Although we were unable to estimate the duration and intensity of disturbance in different sites, the occurrence of most sampled sites, either close to the coast or roadside, indicated some sort of disturbance. Thus, human‐induced disturbance appears as one of the most important factors for the establishment and invasion of *H. persicum,* as predicted for successful invaders in the cold environment (Lembrechts et al., 2016), and the presence of *A. sylvestris* may be taken as an indication that the habitat is invasible by *H. persicum*.

## 5 CONCLUSION

Our results clearly show that invasion by *H. persicum* exerts strong negative pressure on native abundance and diversity. We further note that the presence of *H. persicum* reduces the vulnerability of plant communities to further invasion. Our results indicate community stability or saturation as an important determinant of invasibility. There was a positive association between genetic diversity of *H. persicum* and invasibility; however, we interpret the invasion history of *H. persicum* as one of the important factors that shape the invasibility rather than the genetic diversity per se. Although there is a trade‐off between invasion resistance and vulnerability to local extinction of native species as a consequence of *H. persicum* invasion, it would be unwise to risk the extinction of native species at the expense of any invasion resistance it may offer.

## CONFLICT OF INTEREST

None declared.
