# Supplementary figures and images for: Giant invasive Heracleum persicum: Friend or foe of plant diversity?
Source: Ecol Evol. 2017 May 30;7(13):4936–50. doi: 10.1002/ece3.3055 (PMC5496559; doi:10.1002/ece3.3055)

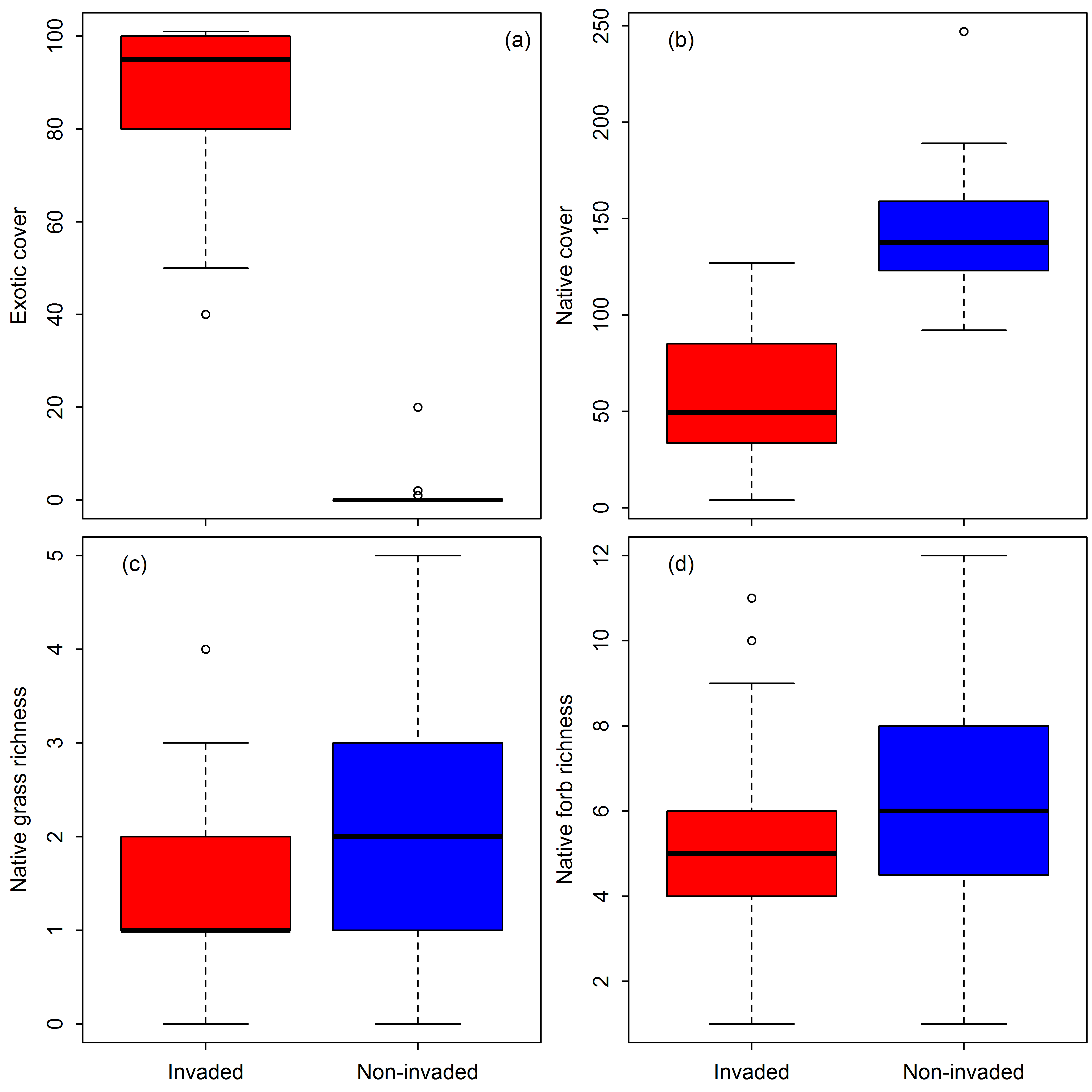

Supplement: Supplementary file 1 [file ECE3-7-4936-s001.tiff]
